# Radiation-Activated Cobalt-Based Zeolite Imidazolate Frameworks for Tumor Multitherapy

**DOI:** 10.34133/bmr.0164

**Published:** 2025-04-15

**Authors:** Qijun Du, Hongwei Jiang, Di Wu, Changlong Song, Wenqi Hu, Qinrui Lu, Chenwei Sun, Jie Liu, Guohua Wu, Shuqi Wang

**Affiliations:** ^1^ Clinical Research Center for Respiratory Disease, West China Hospital, Sichuan University, Chengdu 610065, China.; ^2^ College of Biomedical Engineering, Sichuan University, Chengdu 610065, China.; ^3^ Luoyang Key Laboratory of Clinical Multiomics and Translational Medicine, Henan Key Laboratory of Rare Diseases, Endocrinology and Metabolism Center, The First Affiliated Hospital, and College of Clinical Medicine, Henan University of Science and Technology, Luoyang 471003, China.; ^4^ Tianfu Jincheng Laboratory, City of Future Medicine, Chengdu 641400, China.; ^5^ National Engineering Research Center for Biomaterials, Sichuan University, Chengdu 610065, China.

## Abstract

Radiation dynamic therapy (RDT) is known to induce cancer apoptosis and death with minimal side effects and high accuracy. However, low efficiency of radiation sensitization and persistent hypoxic environment in tumors pose marked challenges for successful RDT. To address these challenges, a novel biodegradable drug delivery system was developed, using quercetin and sorafenib-loaded ZIF67 nanoparticles (QSZP NPs) coated with polydopamine. This system effectively controlled the tumor microenvironment (TME), overcame hypoxia, and was thus utilized for collaborative RDT and radiotherapy (RT). The QSZP NPs demonstrated great potential in x-ray sensitization and reactive oxygen species (ROS)-mediated effects in vitro. Furthermore, they continuously generated oxygen and increased ROS levels in the TME with x-ray irradiation to achieve RDT. In vivo studies showed that QSZP NPs had no apparent systemic toxicity and showed good therapeutic effect in a HepG2 tumor-bearing model. Due to its unique and outstanding combinational effect of RDT/RT/antiangiogenic cancer therapy, these synthesized NPs offer a promising method for radiation-based cancer treatment.

## Introduction

Radiotherapy (RT) is a primary clinical treatment for tumor, utilizing high-energy ionizing radiation to induce DNA damage and apoptosis in tumor cells [1,2]. However, conventional RT is influenced by factors in the tumor microenvironment (TME), such as acidic pH, overexpression of vascular endothelial growth factor (VEGF), and hypoxia, which may limit its therapeutic efficacy [3–5]. Recent studies have shown that reactive oxygen species (ROS) are produced under x-ray irradiation with lethal toxicity, known as radiation dynamic therapy (RDT) [6,7]. These ROS can spread within the tumor region and effectively kill cancer cells.

Recent years have seen a surge in interest in strategies aimed at improving tumor reoxygenation to enhance the efficacy of RT. These strategies primarily include O_3_ therapy, hyperbaric oxygen therapy, and oxygen-carrying nanocarriers [8–10]. zeolitic imidazolate framework-90 is a highly oxygen-carrying metal-organic framework (MOF) material that has a specific response to adenosine triphosphate, which is highly expressed in the mitochondria of cancer cells [11]. Chen et al. [12] catalyzed H_2_O_2_ to produce ·OH for enhanced RT using manganese dioxide nanoparticles. The O_3_ therapy mainly uses O_3_ to enhance the oxygen concentration in TME and accelerate the reoxidation rate. Cu^2+^ NCs can catalyze H_2_O_2_ in the cell to produce active ·OH, which destroys cancer cells through Haber-Weiss reactions [13]. Elements in various redoxic states, such as cobalt (Co) and chromium (Cr), have also been shown to convert intracellular H_2_O_2_ to toxic ·OH through Fenton-like reactions, but have not been widely used for tumor radiosensitization [14–16]. Therefore, the development of a novel multifunctional RT sensitizer with excellent biocompatibility to enhance the ROS-mediated therapy is urgently required to optimize treatment outcomes.

MOFs are emerging as promising porous nanomaterials with unique characteristics such as strong chemical bonds between metal-containing units and organic ligands through self-assembly [17–19]. They offer tailorable pore structures, abundant channels, and good degradability and have become an attractive nanocarrier platform for tumor therapy [20,21]. Zeolitic imidazolate framework (ZIF67) materials with biomacromolecules have been demonstrated to be effective biological application vectors [22,23]. Additionally, ZIF67 derivatives can serve as electromagnetic absorption materials and H_2_O_2_ catalysts [24–26]. Despite recent progress in MOF-based cancer therapy, the effectiveness of RT is markedly hindered by the complex characteristics of the TME, particularly hypoxia [27–29]. However, the existing nanodelivery systems that incorporate enzymes to alleviate hypoxia are overly complex. Their design must take into account the specific cavity properties that correspond to the enzymes, which markedly restricts their broader application in therapeutic settings [29]. Therefore, both basic and clinical research is necessary to develop multifunctional MOF platforms in conjunction with RDT to achieve better overall outcomes in tumor treatment.

**Fig. 1.**
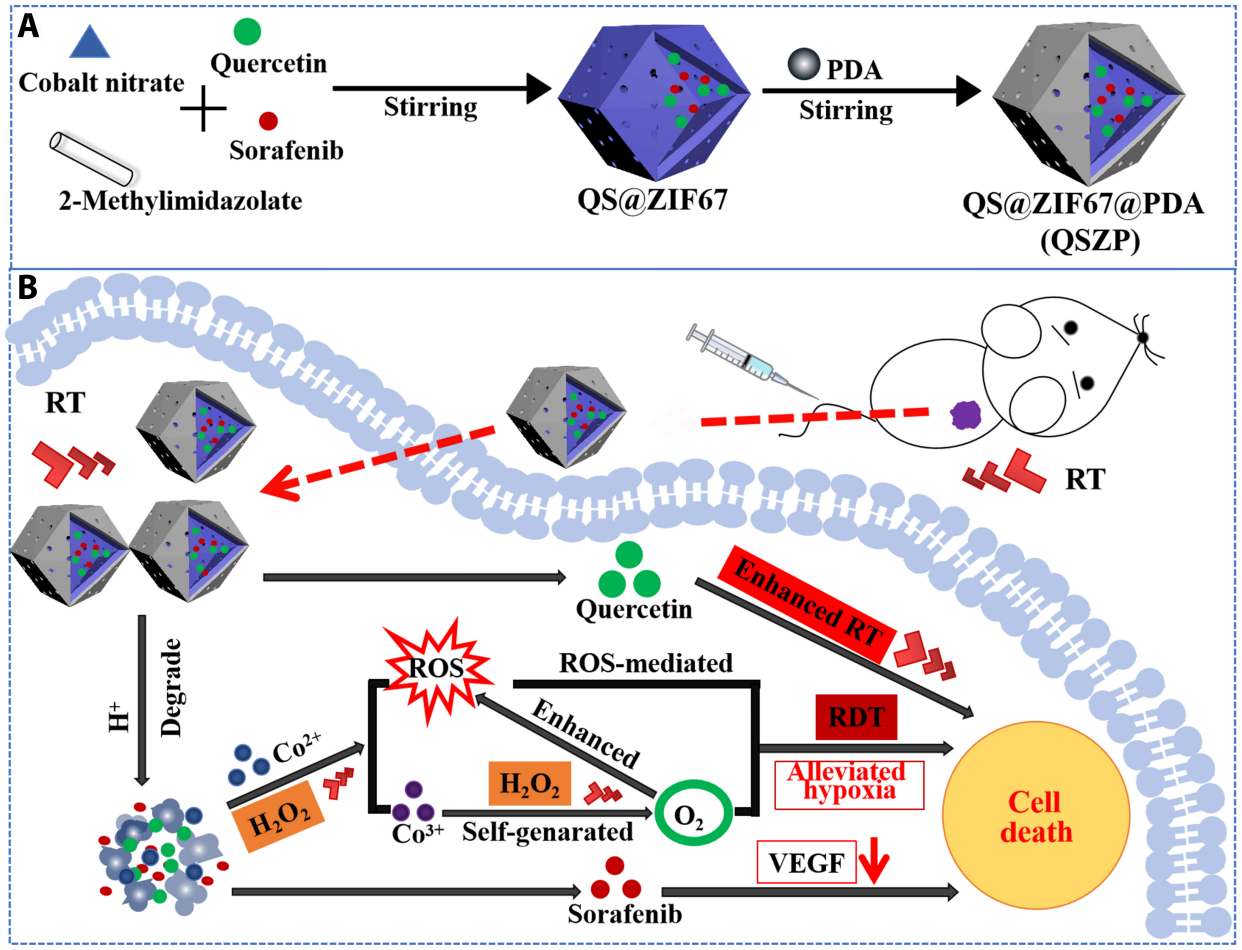
(A) Synthetic schematic diagram of QSZP NPs. (B) The antiangiogenesis-RT synergistic therapy of QSZP NPs can produce oxygen to enhance the generation of ROS for improved RDT therapy and induced cell death.

Herein, we have designed a multiresponsive biodegradable radiosensitizer comprising quercetin@sorafenib@ZIF67 NPs modified with polydopamine (PDA) to simultaneously enhance x-ray absorption energy and to overcome tumor hypoxia (Fig. 1). ZIF67 NPs serve as an efficient catalyst for hydrogen peroxide, catalytically generating oxygen to alleviate tumor hypoxia and demonstrating potential response performance to RT. Quercetin, a radiosensitizer, was loaded with ZIF67 NPs to improve its sensitization effect during RT [30,31]. The antitumor angiogenic drug sorafenib was encapsulated within ZIF67 NPs to enhance its bioavailability [32,33]. The combination of sorafenib with the oxygen boosting therapy provided by ZIF67 NPs improved hypoxia and prevented tumor metastasis and recurrence. To improve the biocompatibility of ZIF67 NPs, their surface was successively coated with PDA, forming a protective shell that circulates throughout the body. This method resulted in multiradiation-responsive synergistic therapeutic NPs capable of achieving synergistic RDT and RT under x-ray irradiation. Within the TME, an acidic degradation response and H_2_O_2_ reaction can occur to release encapsulated sorafenib and to initiate oxygen-enhanced synergistic therapy. The overexpression of H_2_O_2_ in tumors can further enhance the effect of RDT. The QSZP NPs demonstrated enhanced efficacy for RT both in vitro and in vivo. It is reasonable to infer that this oxygen self-supply and drug delivery complex can augment the sensitizing effect of x-ray for clinical treatment of deep-seated tumors.

## Materials and Methods

### Materials

Cobalt nitrate hexahydrate (Co(NO_3_)_2_^.^6H_2_O) and quercetin (Qu) were obtained from Shanghai Macklin Biochemical Co., Ltd. Anhydrous methanol was obtained by Shanghai Titan Technology Co., Ltd. 2-Methylimidazole (2-MI) was acquired from Beijing Bailingwei Technology Co., Ltd. Sorafenib (SR) was purchased from Shanghai Aladdin Chemistry Company. Hydrogen peroxide (H_2_O_2_) and ammonia were provided by Chengdu Colon Chemical Co., Ltd. Dopamine hydrochloride (PDA) was provided by Chengdu McCaxi Chemical Co., Ltd. Cell Counting Kit-8 (CCK-8) was obtained from Tongren Institute of Chemistry. The DNA Damage Assay Kit by γ-H_2_AX immunofluorescence, the ROS detection kit (2,7-dichlorodihydrofluorescein diacetate [DCFH-DA]), and the cell viability/cytotoxicity assay kit were provided by Shanghai Beyotime Biotechnology Co., Ltd.

### Preparation of QS@ZIF-67 NPs

Firstly, 5 mg of quercetin and 5 mg of sorafenib were weighed and dissolved in 4 ml of methanol. Subsequently, 0.3 ml (50 mg/ml) of a cobalt nitrate methanol solution and 0.9 ml (200 mg/ml) of a 2-MI methanol solution were prepared. The quercetin and sorafenib methanol solution (4 ml) was added to the mixed 2-MI solution, and then stirred at mild conditions for 10 min. The cobalt nitrate methanol solution was then slowly added into the mixed methanol solution of 2-mimethylimidazole. 

After stirring slowly for 6 h, the quercetin@sorafenib@ZIF67 (QS@ZIF67) NPs were obtained via centrifugation (13,000 rpm, 4 min), and then washed 3 times with methanol for further use.

### Preparation of QSZP NPs

In a typical system, 40 mg of the QS@ZIF67 NPs was dispersed into 3 ml of methanol. Dopamine hydrochloride (5 mg) and 50 μl of ammonia (NH_3_^.^H_2_O, 25% to 28%) were dispersed into 1 ml of methanol, slowly added into the above solution, and then stirred for 1 h. The QSZP NPs were obtained via centrifugation (13,000 rpm, 4 min) and then washed with dH_2_O for further use. Quercetin@ZIF67@PDA (QZP) NPs were obtained in the absence of SR during the preparation process described above. Similarly, sorafenib@ZIF67@PDA (SZP) NPs were obtained without the addition of quercetin.

### Degradation of QSZP NPs

QSZP NPs (0.5 mg/ml) is dispersed in phosphate buffered saline (PBS, pH 5.7 or pH 7.4) and shaken in a water bath at 37 °C. Equal volumes of QSZP NPs were collected for scanning electron microscopy (SEM) analysis to assess degradation over time.

### ROS test in vitro

ROS formation was characterized by using the DCFH-DA method. Grouping was as follows: PBS (defined as the negative control group), PBS + RT (only x-ray irradiation), PBS + QSZP, and PBS + QSZ + RT. Specifically, a DCFH solution (10 μl) was added to the PBS (pH 5.7) dispersion of the QSZP NPs at a concentration of 5 mg/ml, respectively. The mixed solution was placed under x-ray irradiation (2 Gy) or non-x-ray irradiation. After x-ray irradiation, keeping for 2 h in the above conditions, the upper solutions were centrifuged (12,000 rpm, 5 min) for subsequent testing of DCF fluorescence. The capacity of ROS production at different conditions was compared at the peak of 520 nm. In addition, the amount of ROS in the presence of H_2_O_2_ was also compared. The samples were divided into 4 groups: PBS + H_2_O_2_, PBS + H_2_O_2_ + RT, PBS + H_2_O_2_ + QSZP, and PBS + H_2_O_2_ + QSZP + RT. A H_2_O_2_ solution of 3.7 mg/ml and QSZP NPs solution of 5 mg/ml were used in the experiments.

### SR release in QSZP NPs

The drug release test was conducted in 2 different PBS buffer solutions. QSZP NPs (100 mg) were immersed in 200 ml of PBS solution at pH 7.4 or pH 5.7 and placed in a shaker incubator at 37 °C. At predetermined time intervals, 2 ml of the release solution was withdrawn and replaced with an equal volume of fresh buffer. The supernatant was then obtained by centrifugation (13,000 rpm for 4 min) and measured at the peak wavelength of 266 nm using an ultraviolet–visible (UV–Vis) spectrophotometer. A standard curve was established by measuring the UV absorption intensity of different concentrations of SR. According to the following formulas, the drug loading contents = (the drug loaded in NPs weight)/(total NPs weight) × 100%. The drug loading efficiency = (total nanoparticles weight × LC)/(total drug weight) × 100%.

### In vitro evaluation of cytotoxicity and therapeutic efficacy

The cytotoxicity of QSZP NPs was assessed by measuring cell activity using CCK-8 assays. HepG2 cells were individually seeded into 96-well plates (5 × 10^3^ cells/well). QSZP NPs at varying concentrations (ranging from 12.5 to 200 μg/ml) were added to the above well and cocultured for 24 h. Cells without NPs served as a control group. Subsequently, CCK-8 solution was added to each well and placed at 37 °C for 1 h. Finally, the absorbance at 450 nm was measured using TECAN M200.

The efficacy of cellular-level therapy was also assessed through the CCK-8 assay, which involved 9 groups: control group, QZP group, SZP group, QSZP group, PBS + RT group, Qu + RT group, QZP + RT group, SZP + RT group, and QSZP + RT group. HepG2 cells were seeded into a 6-well plate at a density of 5 × 10^5^ cells/well. The culture medium or a medium solution containing 100 μg/ml of Qu/QZP/SZP/QSZP NPs was added to the wells, respectively. After coincubation for 24 h, the groups containing RT were exposed to stimulation by x-ray irradiation at a dose of 2 Gy. The cells were incubated further for 12 h before CCK-8 detection. Furthermore, Calcein-AM/PI staining was used to characterize the condition of living and dead cells under different treatment. After the above similar treatment, the cells were washed, then added with Calcein-AM and PI, and incubated for 10 min under gentle shaking. Finally, the fluorescence of the cells was observed under inverted fluorescence microscope.

### Evaluation of ROS at a cellular level

ROS production in HepG2 cells was detected by DCFH-DA probe. Firstly, 5 × 10^5^ HepG2 cells were seeded into 6-well plates and randomly divided into 4 groups: control group, QZSP group, PBS + RT group, and QSZP + RT group. The therapeutic concentration of QSZP NPs was 100 μg/ml and the x-ray power was 2 Gy. Then, removing the solution after 24 h of coculture, 200 μl of Dulbecco’s modified Eagle’s medium (including 1 μM DCFH-DA) was added, and x-ray irradiation was performed. Finally, fluorescence was observed under an inverted fluorescence microscope.

### γ-H_2_AX immunofluorescence analysis in cells

The experiment comprised 4 groups: control group, QSZP group, RT group, and QSZP+RT group. HepG2 cells (5 × 10^5^ cell/well) were seeded in 6-well plates. After adding QSZP NPs at a concentration of 100 μg/ml for 24 h, the groups receiving RT were irradiated at 2 Gy. Subsequently, the medium was removed, and the cells were washed with PBS. The cells were fixed and incubated for 10 min, followed by washes with detergent. Immunostaining blocking solution was added and incubated for 10 min at room temperature. After removing the blocking solution, the cells were incubated with γ-H_2_AX monoclonal antibody overnight at 4 °C. The cells were washed 3 times for 5 min each, followed by incubation with anti-rabbit 488 for 1 h at room temperature. The cells were washed twice for 5 min each, and then stained with nucleus staining solution (DAPI) for 5 min at room temperature. After removing the stain, the cells were washed 3 times for 5 min each. Finally, the cells were analyzed using an inverted fluorescence microscope.

### In vivo therapy experiment

The animal experiments were conducted following the experimental protocols approved by the Sichuan Kangcheng Biotechnology Co. Ltd. (authorization number 2022-KM-M-012-010). For the antitumor efficacy in vivo, HepG2 tumor-bearing mice with a tumor volume of 160 ± 20 mm^3^ were selected as the experimental model. Female Balb/c nude mice (approximately 19 ± 2 g) were purchased from Beijing Vital River Laboratory Animal Technology Co., Ltd. Tumor-bearing mice were randomly allocated into 6 groups (3 mice in each group): control group, Qu + SR + RT group, QSZP group, QZP + RT group, SZP + RT group, and QSZP + RT group. The NPs (100 mg/kg) were intravenously injected into mice, respectively. Six hours following injection, the tumor site of mice in groups were treated with x-ray irradiation (5 Gy). The tumor volume and body weight were assessed during treatment. The tumor volume was calculated as follows: tumor volume = long diameter^2^ × long diameter/2. The tumor diameter was measured using Vernier calipers. On the 25th day, the mice from all groups were sacrificed, and the tumor, heart, liver, spleen, lung, and kidney were dissected for further systematic pathological assessment.

To further confirm the successful generation of ROS in vivo and their role in mediating RT, tumor-bearing mice were sacrificed after the respective treatments, and the tumors were harvested for cryosectioning. The tumor sections were then fixed with acetone at 4 °C and washed with PBS. Following this, the sections were incubated with the ROS probe DCFH-DA at 37 °C for 1 h, washed 3 times with PBS, and stained with DAPI for 10 min to obtain the final fluorescence images.

To evaluate the potential toxicity of QSZP, tumor-free Balb/c mice were administered a single dose of PBS (100 mg/kg) via tail vein injection. Blood was collected directly from the hearts of the mice 45 days postinjection. The blood samples were then centrifuged at 3,000 rpm for 10 min to separate the plasma for the analysis of aspartate aminotransferase (AST) and alanine aminotransferase (ALT) levels. Finally, the hearts, livers, spleens, lungs, and kidneys were harvested, embedded in paraffin, sectioned to a thickness of 5 μm, and analyzed using hematoxylin and eosin (H&E) staining.

### Statistical analysis

The experimental results are expressed in the form of mean ± standard deviation. Data were analyzed by a one-way analysis of variance followed by Tukey’s multiple comparison test using SPSS 22.0 software. The statistical value of the experimental results is <0.05, indicating statistical significance. *P < 0.05, **P < 0.01.

## Results and Discussion

**Fig. 2.**
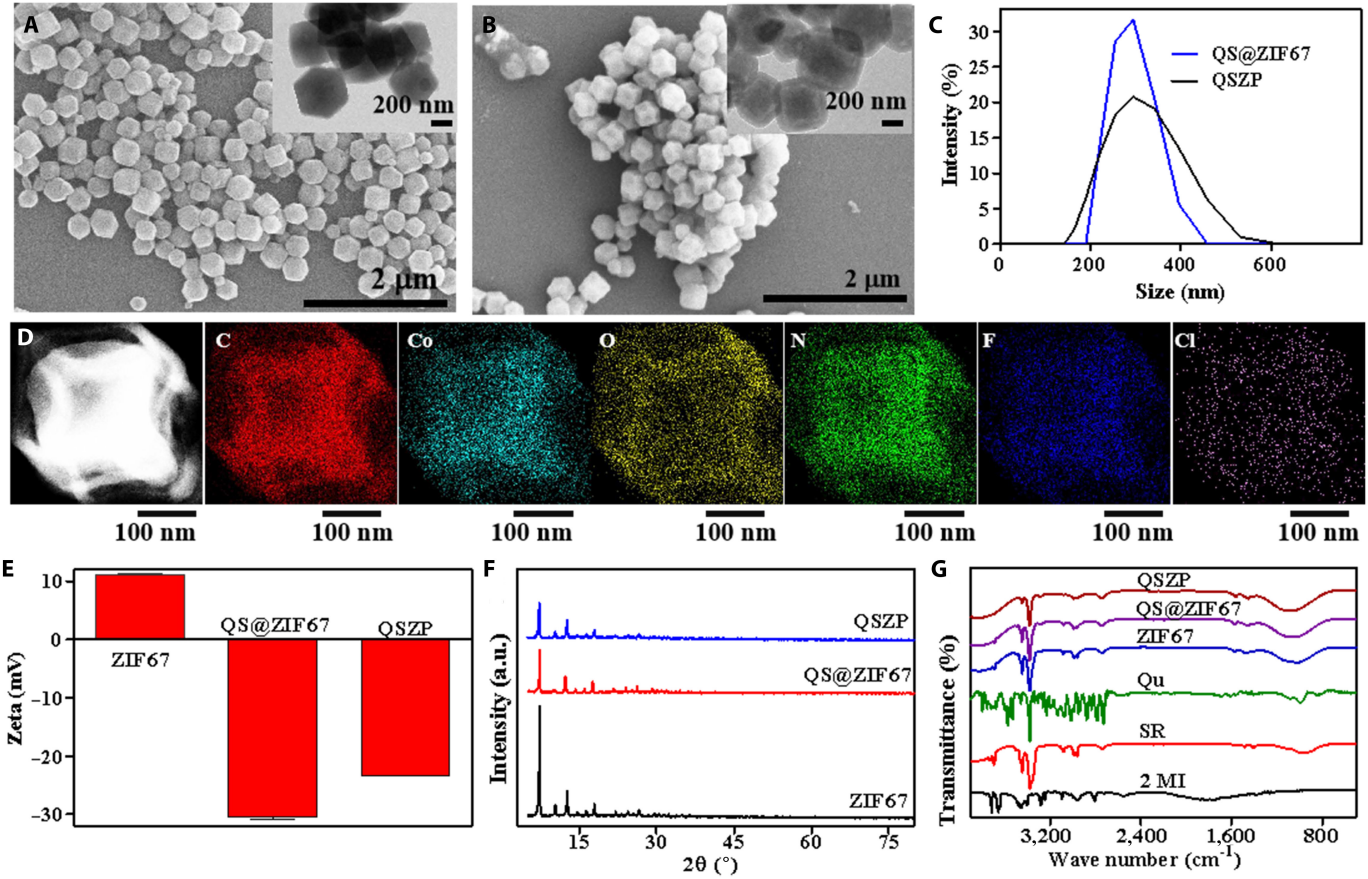
Characterization of the QSZP NPs. (A and B) The SEM image of QS@ZIF-67, QSZP NPs. The inset is its TEM image. (C) Dynamic light scattering characterization of NPs from (A) and (B). (D) Dark-field TEM image and mapping image of QSZP NPs. (E) Zeta potential of the ZIF-67, QS@ZIF67, and QSZP NPs. (F) X-ray diffraction (XRD) analysis of ZIF-67, QS@ZIF67, and QSZP NPs. (G) The Fourier transform infrared spectroscopy (FT-IR) spectra of 2 MI, SR, Qu, ZIF-67, QS@ZIF-67, and QSZP NPs.

The synthetic process is presented in Fig. 1. QSZP NPs were prepared via stirring at room temperature and subsequently coated with PDA to lower the cytotoxicity and to enhance biosafety. Additionally, PDA was used to increase drug release and biodegradation in vivo, thus increasing the efficacy of tumor therapy. The synthesized QS@ZIF67 NPs have a solid dodecahedral crystal structure with obvious corner angle (Fig. 2A), and its hydrated particle was 252.48 nm (Fig. 2C). As presented in Fig. 2B, it can be observed that QS@ZIF67 NPs were wrapped by a PDA shell, and the size of hydrated QSZP NPs is as high as 304.04 nm (Fig. 2C). Moreover, Fig. 2D illustrates the mapping results of QSZP NPs, confirming the distribution of elements including C, Co, O, N, F, and Cl. The presence of F and Cl elements in the mapping results can be attributed to SR. Subsequently, QSZP NPs were characterized by energy dispersive x-ray spectroscopy. As can be seen from Fig. S1, the relevant elemental features include C, Co, O, N, F, and Cl. Additionally, the Zeta potential values of ZIF67, QS@ZIF67, and QSZP NPs were 11.13, −30.47, and −23.33 mV, respectively (Fig. 2E).

In addition, XRD, Fourier transform infrared spectroscopy (FT-IR), and thermogravimetry (TG) were performed to further characterize the QSZP NPs. As shown in Fig. 2F, the peak positions of ZIF67 NPs, QS@ZIF67, and QSZP NPs were essentially identical, indicating that the crystal morphology of ZIF-67 remained unchanged after modification. FT-IR was likewise applied to certify the presence of quercetin, SR, and PDA within the system. ZIF67 was characterized by the bending vibration peak of -CH_3_ at 1,384 cm^−1^ and the bending vibration peak of C-N at 1,141 cm^−1^ (Fig. 2G). Following encapsulation of quercetin and SR, the C=O stretching vibration peak was observed at 1,654 cm^−1^. Following coating with PDA, a phenolic bending vibration peak was observed at 1,302 cm^−1^. Comparing with the spectrum of ZIF67, QU and BSA were successfully loaded and attached on the ZIF67. Comparing with the spectrum of ZIF67, quercetin, SR, and PDA were successfully loaded and attached to the ZIF67. The mass loadings of Qu within the QSZP NPs are determined to be 9.49% by TG (Fig. S2). Altogether, QSZP NPs were successfully synthesized and characterized.

Drug-loaded nanoplatforms that are pH responsive and tumor targets under the conditions of the TME can markedly enhance the therapeutic efficacy of drugs. Meanwhile, the drug decomposes slowly under neutral conditions, thereby reducing its overall toxic and side effects in vivo. Only in a weak acidic environment is the MOF structure of QSZP NPs destroyed, releasing Co (II), and achieving catalase-like and peroxide-like activities. Neutral PBS solution with a pH of 7.4 was used to mimic the physiological environment, while weakly acidic PBS solution with a pH of 5.7 was used to simulate the TME for studying the degradation characteristics of QSZP NPs. As shown in Fig. S3, QSZP NPs degraded slowly under neutral conditions, so that its morphology remained unchanged after soaking for 24 h. After 3 h of coincubation in weakly acidic PBS solution, QSZP NPs maintained their shape, while most of QSZP NPs degraded after 6 h. Especially after 24 h, QSZP NPs basically completely degraded (only the PDA shell remained). Following intravenous injection into mice via the tail vein, the MOF structure of the QSZP NPs was disrupted under slightly acidic conditions, which triggered the release of both Co(II) ions and the antiangiogenic agent SR, both exhibiting peroxidase-like activity.

**Fig. 3.**
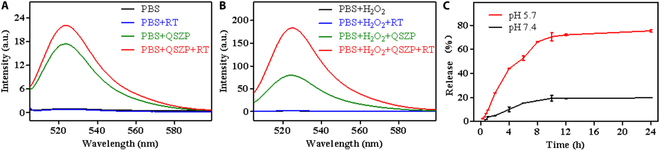
Evaluation of ROS production capacity and drug release. (A) The results of QSZP NPs to generate ROS under x-ray irradiation. (B) The effect of QSZP NPs to generate ROS under non-x-ray irradiation, x-ray irradiation, and exposure to H_2_O_2_, respectively. (C) Release results of sorafenib in QSZP NPs under neutral and weakly acidic PBS (*n* = 3).

Cytotoxic ROS have the ability to eliminate cancer cells by triggering autophagy and apoptosis. The rapid metabolism of cancer cells and inadequate blood supply can lead to overexpression of H_2_O_2_ levels within the tumor. Therefore, converting H_2_O_2_ into toxic ROS by using catalyst under x-ray irradiation is an effective way to improve the sensitivity of RT. ROS can change DCFH-DA into 2,7-dichlorofluorescin, which has evident green fluorescence (λex = 488 nm and λem = 525 nm). Under weakly acidic conditions, the MOF structure of QSZP NCs was destroyed, leading to the release of Co (II) with peroxidase activity. Subsequently, cobalt (II) can catalyze the disproportionation of H_2_O_2_ to generate highly oxidizing hydroxyl radicals (•OH) and cobalt (III), while cobalt (II) facilitates the production of ROS by promoting the conversion of H_2_O_2_ to O_2_ in the tumor. As shown in Fig. 3A, it can be observed that there is minimal ROS generation in both the PBS group and the PBS + RT group. After adding QSZP NPs to the PBS solution, a considerable amount of ROS was generated in the solution. The ROS content produced through QSZP NPs under x-ray irradiation was 1.27 times larger than that produced by the single QSZP NPs. Since the TME contains a large amount of H_2_O_2_, the effect of high concentration of H_2_O_2_ on ROS generation was further investigated. As presented in Fig. 3B, it is evident that there is minimal ROS generation in the PBS + H_2_O_2_ and PBS + H_2_O_2_ + RT groups. However, upon introducing QSZP NPs into PBS, a considerable amount of ROS is generated in the solution. Furthermore, the combination of x-ray with QSZP NPs can enhance the decomposition of H_2_O_2_ into O_2_, leading to a substantial increase in ROS levels in the solution. Consequently, the ROS concentration in the PBS + H_2_O_2_ + QSZP + RT group is 2.29 times higher than that in the PBS + H_2_O_2_ + QSZP group. Clearly, the catalytic effect of QSZP NPs combined with x-ray promotes the conversion of H_2_O_2_ to O_2_, thereby enhancing ROS generation in RDT and facilitating the realization of RDT.

Since QSZP NPs can be degraded in a simulated TME, we hypothesized that QSZP NPs would be suitable as antitumor drug carriers to achieve controlled release in the TME. SR is a widely applied, primary antiangiogenic therapy drug for tumor treatment able to down-regulate the expression of VEGF. SR was loaded into the mesoporous structure of QSZP NPs to combine RDT with antiangiogenic. The encapsulation efficiency of SR was determined by UV–Vis spectrophotometry. The loading efficiency of SR in QSZP NPs was 18.32%, while the encapsulation efficiency was 30.37%. As illustrated in Fig. 3C, the release rates of SR from QSZP NPs were 19.77% at a pH of 7.4 and 75.37% at a pH of 5.7. The release rate of SR in weak acidic PBS was 3.81 times higher than that in neutral PBS. The rapid release of SR in weak acidic conditions is attributed to the disintegration of the MOF structure of QSZP NPs, which accelerates the release process. These findings indicate that the pH-responsive release of QSZP NPs can effectively achieve a controlled release of SR, thereby inhibiting tumor angiogenesis following RT/RDT therapy.

**Fig. 4.**
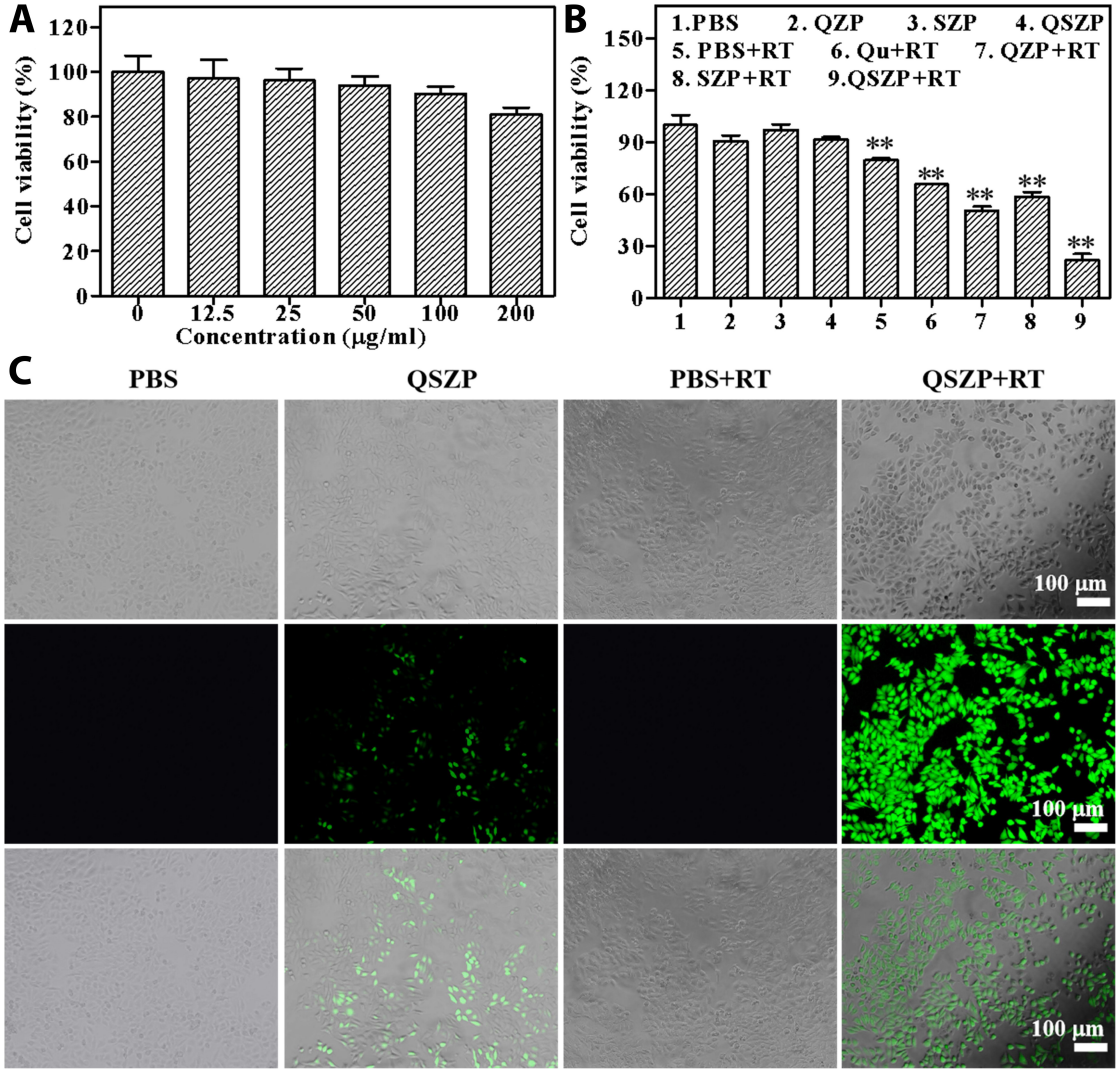
Evaluation of QSZP NPs biocompatibility, antitumor efficacy, and intracellular ROS generation. (A) The cell viability of HepG2 cells incubated with QSZP NPs (*n* = 3). (B) The cell viability of HepG2 cells under different treatments. (C) The results of QSZP NPs to generate ROS under x-ray irradiation. **P < 0.01, versus the PBS group.

Biocompatibility is of major importance for biomedical applications. Cell viability testing was performed to investigate the biosafety of QSZP NPs. Firstly, HepG2 cells were cocultured with different concentrations of QSZP NPs for 24 h. Then, CCK-8 was added to verify cell viability. As presented in Fig. 4A, the viability of HepG2 cells was 81.08%, when treated with QSZP (200 μg/ml). The high viability suggested that QSZP NPs have excellent biocompatibility and can be used for antitumor research in vivo. After that, the viability of HepG2 tumor cells treated with RT combined with SR was assessed using CCK-8 assay and Calcein-AM/PI staining. As shown in Fig. 4B, the cell activity of QZP, SZP, and QSZP groups was similar to that of the control group at a concentration of 100 μg/ml, indicating that the QZP, SZP, and QSZP groups had good biocompatibility. In contrast, QZP + RT and SZP + RT groups showed marked tumor inhibition, confirming the dynamic effects of QZP- and SZP-mediated RT. In addition, the QSZP + RT group exhibited the lowest cell viability and the most pronounced red fluorescence of dead cells (Fig. S4), indicating that the combined effect of the tumor angiogenesis inhibitor and RT sensitization markedly enhanced the therapeutic efficacy against tumor cells. Thus, the enhanced tumor cell killing efficiency is attributed to the radiosensitizing effect of a specific concentration of QSZP NPs under x-ray radiation, as well as the inhibitory action of SR on tumor VEGF expression.

After confirming the effectiveness of RT, the ROS mediated by QSZP NPs at the cellular level under x-ray irradiation was evaluated. In this study, DCFH-DA was used as a fluorescent probe for ROS. HepG2 cells were treated with QSZP NPs for 24 h, followed by the addition of culture medium containing DCFH-DA and x-ray irradiation. As shown in Fig. 4C, fluorescence was not observed in the control group or the RT group, indicative of no ROS production in these groups. However, fluorescence was detected in the cells treated with QSZP NPs, suggesting the presence of low levels of ROS captured by the DCFH-DA probe. Interestingly, the fluorescence intensity was higher in the QSZP + RT group after x-ray irradiation, indicating a marked increase in ROS production in the cells under the combined effect of x-ray irradiation and QSZP NPs. Therefore, upon entering the TME, QSZP NPs can enhance intracellular ROS production under x-ray irradiation.

DNA double-strand breaks are one of the most severe forms of DNA damage and can be utilized to assess the biological effects of RT. Upon occurrence of a double-strand break, serine 139 on H_2_AX undergoes phosphorylation (γ-H_2_AX). Therefore, the level of γ-H_2_AX can reflect the degree of DNA damage and is widely used in DNA damage research. In order to evaluate the degree of DNA damage induced by RT/RDT under different treatments in HepG2 cells, the formation of DSB was visualized by γ-H_2_AX immunostaining. As shown in Figs. S5 and S6, in the absence of x-ray irradiation, the fluorescence intensity of γ-H_2_AX in PBS and QSZP groups was weak, indicating no obvious DNA damage. Furthermore, the fluorescence intensity of γ-H_2_AX in the QSZP + RT group was 5.50 times higher than that in the PBS + RT group under x-ray irradiation, which may be the result of the coordination of RDT and RT, since the production of ROS was enhanced and the release of loaded drugs was promoted.

**Fig. 5.**
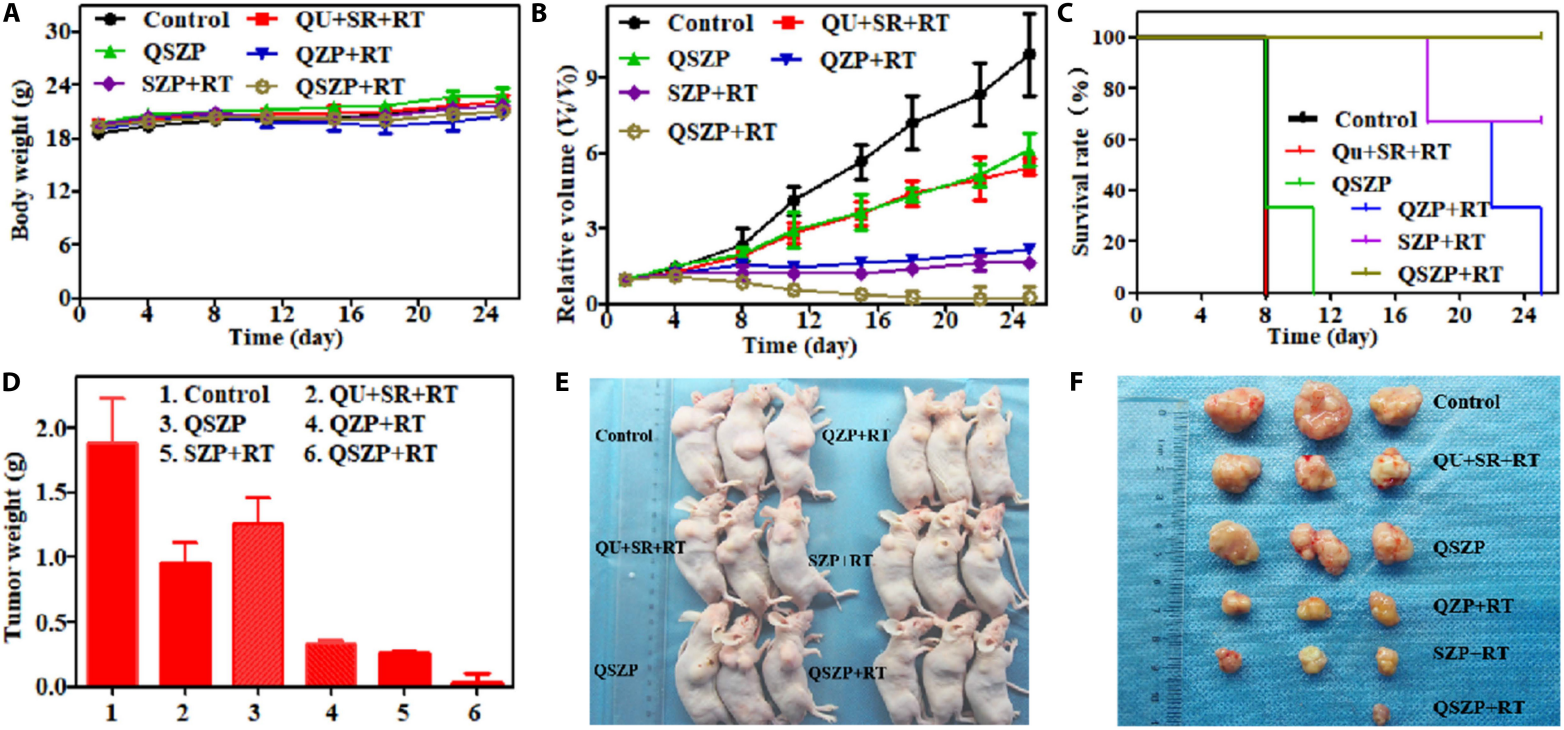
Evaluation of the therapeutic effect of QSZP NPs in vivo (3 mice in each group). (A and B) The curve of weight change and tumor volume changes in balb/c nude mice within 25 days after different treatments. (C) Survival curves of different treatment groups. (D) Tumor weight of balb/c nude mice in each group. (E) Representative photographs of mice bearing HepG2 tumors after various different treatments. (F) Photographs of tumor tissue after excision in different treatment groups.

**Fig. 6.**
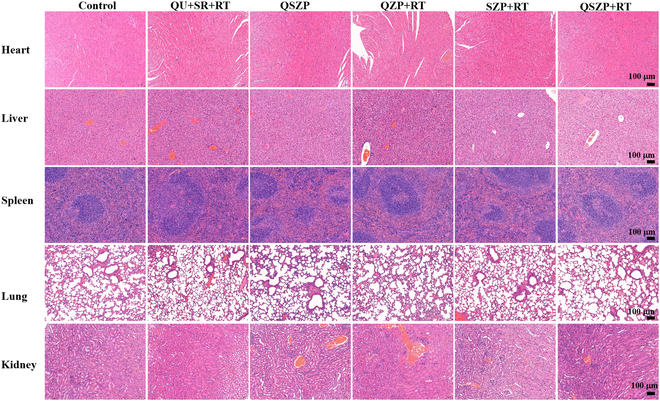
H&E staining images of the major tissues including heart, liver, spleen, lung, and kidney from the mice of different groups.

**Fig. 7.**
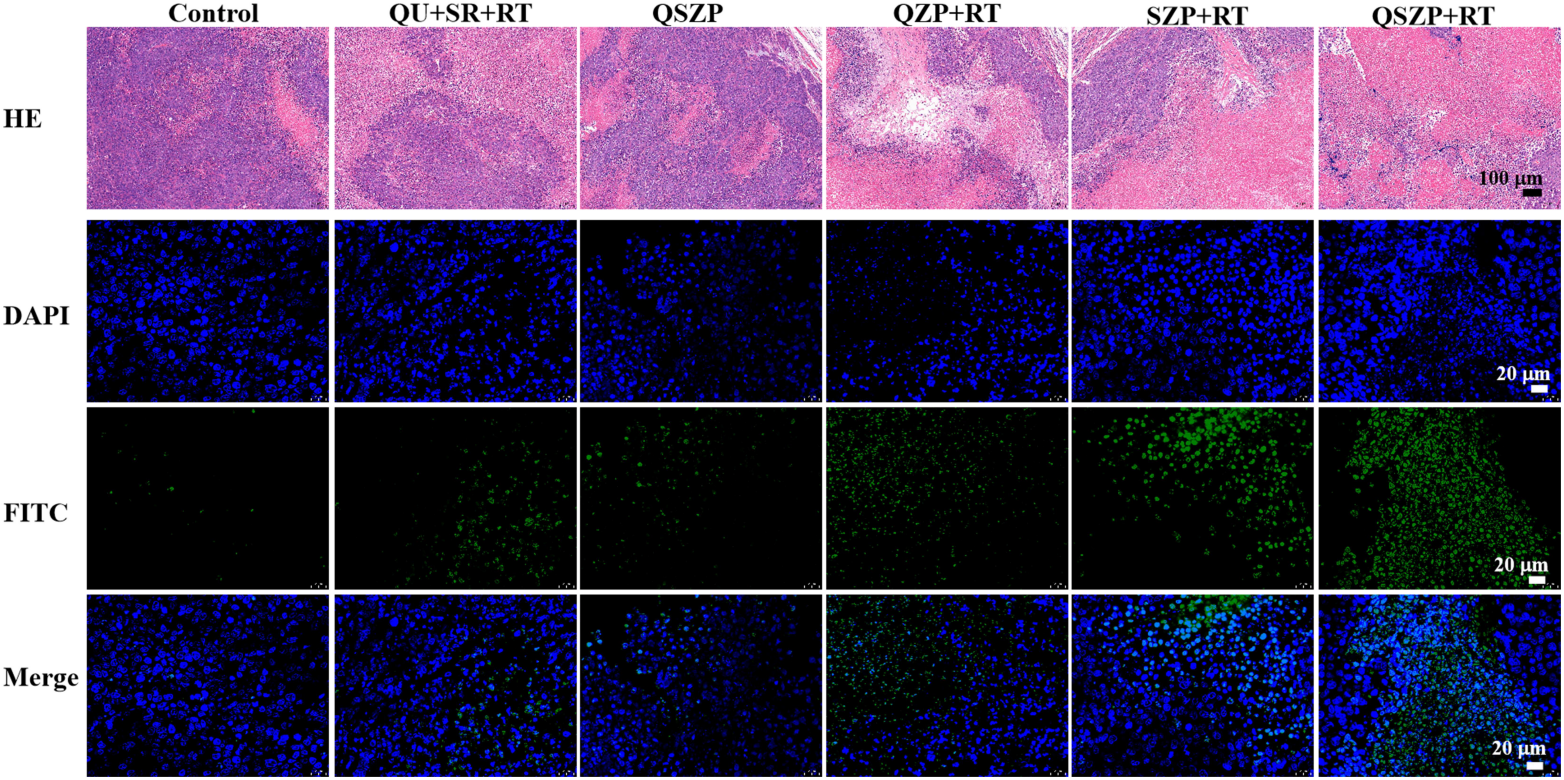
H&E and TUNEL staining of the excised tumors from the mice at 25 days after various different treatments.

The above experiments showed that the synthesized QSZP NPs had enormous probability for inhibiting the cytoactivity of tumor cells in vivo. In order to research the inhibitory effects in vivo, HepG2-bearing mice were randomly divided into a control group, Qu + SR + RT group, QSZP group, QZP + RT group, SZP + RT group, and QSZP + RT group. Six hours following the injection of mice in the Qu + SR + RT group, QZP + RT group, SZP + RT group, and QSZP + RT group, mice were exposed to x-ray (5 Gy). The body weight of mice and tumor volume were measured within 25 days after treatment. The average body weight of all 6 groups were gradually increased (Fig. 5A and Table S1). The tumor volume changes from 0 to 25 days were recorded (Fig. 5B). Compared to the control group, the tumor volume was markedly reduced in the other groups. Furthermore, the QSZP + RT group had a smaller tumor volume compared to the QZP + RT group and the SZP + RT group. In the experiment, we determined that mice were considered sacrificed when the tumor volume exceeded 300 mm^3^. The survival curve also indicated that the QSZP + RT group exhibited the highest antitumor efficacy at 100% (Fig. 5C). Figure 5D and Fig. S7 demonstrated tumor weight and size; the tumor inhibition rate of mice in the QSZP + RT group was 98.03%, which was markedly higher than that of mice in the QZP + RT (82.25%) and QSP + RT (86.18%) groups. Representative images of tumor tissue and mice also verify that the QSZP + RT group had the highest antitumor efficiency (Fig. 5E and F). At the same time, the H&E staining results of the major organs in Balb/c nude mice indicated that QSZP NPs did not cause any noticeable damage to tissues or organs, demonstrating the good biosafety of QSZP NPs (Fig. 6). The H&E staining and immunohistochemical analysis of tumor cell apoptosis (terminal deoxynucleotidyl transferase–mediated deoxyuridine triphosphate nick end labeling [TUNEL]) in the treated tumor mice were performed. As presented in Fig. 7 and Fig. S8, the tumors of mice in the SZP + RT and QSZP + RT group had large necrotic areas, whereas these areas were relatively smaller in mice in the other treatment groups. Furthermore, in the tumor tissue sections of the QZP+RT, SZP + RT, and QSZP + RT groups, the green fluorescence signal produced by the oxidation of DCF was more pronounced, indicating that QZP, SZP, and QSZP could successfully generate ROS under radiation exposure, thereby mediating RT (Fig. S9). Meanwhile, the results of ALT, AST, and H&E staining indicated that QSZP nanoparticles exhibit no marked toxicity to tumor-free Balb/c mice (Figs. S10 and S11). The results of in vivo antitumor experiments have shown that QSZP NPs are capable of generating sufficient oxygen to enhance tumor reoxygenation when exposed to x-ray irradiation. This can enhance the efficacy of combined RT and RDT treatment.

Thus, the outstanding treatment effect of QSZP NPs under x-ray irradiation was considered to be due to the following (Fig. 1B): (a) ZIF67 can effectively enhance the targeted delivery of SR and Huperzine A, allowing the drugs to concentrate more effectively at the tumor site. This effect is attributed to the enhanced permeability and retention effect, which improves therapeutic efficacy. Moreover, the nanoparticle structure of ZIF67 increases the uptake of these drugs by tumor cells, ensuring that SR and Huperzine A are absorbed more efficiently. (b) SR, as a multitargeted tyrosine kinase inhibitor, effectively suppresses the proliferation of tumor cells and angiogenesis, while Huperzine A further enhances the efficacy of RT at the tumor site. The combined use of these 2 drugs during RT generates a synergistic effect, increasing the sensitivity of tumor cells and inhibiting the development of drug resistance. (c) The structural design of ZIF67 enables it to release the loaded drugs under specific conditions, such as low pH environments, which are closely related to the acidic characteristics of the TME. This feature facilitates the timed release of drugs at the tumor site, effectively targeting and killing tumor cells. (d) When the ZIF67 core is exposed to x-ray irradiation, it acts as a catalyst to generate hydrogen peroxide (H_2_O_2_), oxygen, and hydroxyl radicals (·OH) within the TME. This process provides a rich source of ROS, overcoming the limitations of RT dynamics and achieving ROS-mediated therapeutic effects. Therefore, the above results showed that the oxygen production of QSZP NPs could be used as an efficient and biocompatible nanodrug for synergistically enhanced anticancer therapy.

## Conclusion

In summary, we have successfully developed multifunctional QSZP NPs as a radiosensitizer for synergistic antiangiogenesis/RT/RDT. The QSZP NPs were synthesized using a simple method, resulting in a high drug-release rate due to their excellent controlled release performance in acidic conditions. ZIF67 is formed by cobalt ions and 2-MI, serves as an efficient catalyst for H_2_O_2_, and catalytically produces oxygen and ·OH to alleviate tumor hypoxia. SR, an antiangiogenic drug, was loaded onto the QSZP NPs to down-regulate the expression of VEGF, which can effectively reinforce RDT. PDA acted as a biocompatible surface layer to relieve cytotoxicity and improve the biosafety of ZIF67 NPs. In vitro and in vivo studies demonstrated that QSZP NPs exhibited excellent radiosensitivity, ROS-mediated effects, and biosafety. Consequently, the tumor inhibition rate of the QSZP NPs under x-ray irradiation was 98.03%. These results may lead to novel synergistic treatment approaches for cancer treatment in clinical settings.

## Data Availability

Data will be made available on request.
