# Supplementary material for: Radiation-Activated Cobalt-Based Zeolite Imidazolate Frameworks for Tumor Multitherapy
Source: Biomater Res. 2025 Apr 15;29:0164. doi: 10.34133/bmr.0164 (PMC11997308; doi:10.34133/bmr.0164)
Supplement: Supplementary 1 — Supplemental Material [file bmr.0164.f1.doc]

**Supplementary Information**

**Radiation-activated Cobalt-based Zeolite Imidazolate Frameworks for Tumor Multi-therapy**

Qijun Du^1,2^, Hongwei Jiang^3^, Di Wu^1,2,4^, Changlong Song^1,2^, Wenqi Hu^1,2^, Qingrui Lu^1,2^, Chenwei Sun^1,2^, Jie Liu^1,2^, Guohua Wu^1,2,3*^, and Shuqi Wang^1,2,4,5*^

^1^Clinical Research Center for Respiratory Disease, West China Hospital, Sichuan University, Chengdu 610065, China. ^2^College of Biomedical Engineering, Sichuan University, Chengdu 610065, China. ^3^Luoyang Key Laboratory of Clinical Multiomics and Translational Medicine, Henan Key Laboratory of Rare Diseases, Endocrinology and Metabolism Center, The First Affiliated Hospital, and College of Clinical Medicine of Henan University of Science and Technology, Luoyang, China, 471003. ^4^Tianfu Jincheng Laboratory, City of Future Medicine, Chengdu 641400, China. ^5^National Engineering Research Center for Biomaterials, Sichuan University, Chengdu 610065, China.

^*^Correspondence: [wuguohua@zju.edu.cn,](mailto:wuguohua@zju.edu.cn,) shuqi@scu.edu.cn.


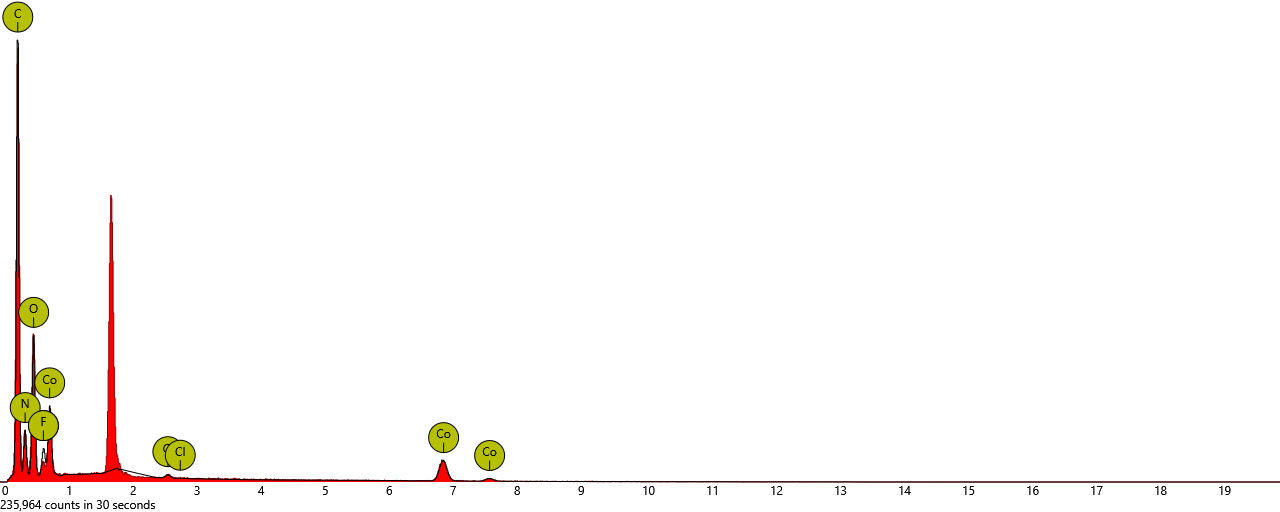


***Fig. S1.*** *The EDS of QSZP NPs.*

Note: The sample is placed on the silicon wafer for detection.


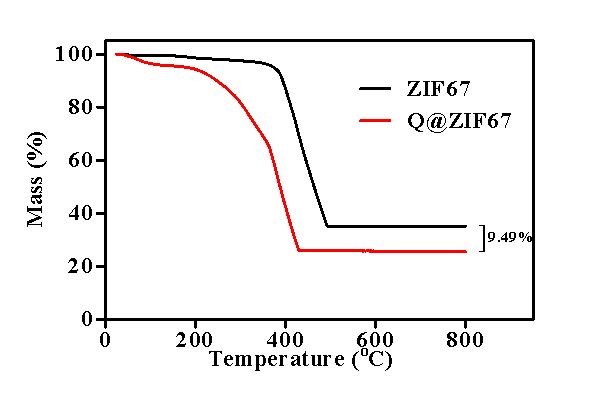


***Fig. S2.*** *Thermo-gravimetry (TG) results of ZIF67 and Q@ZIF67 NPs.*

*
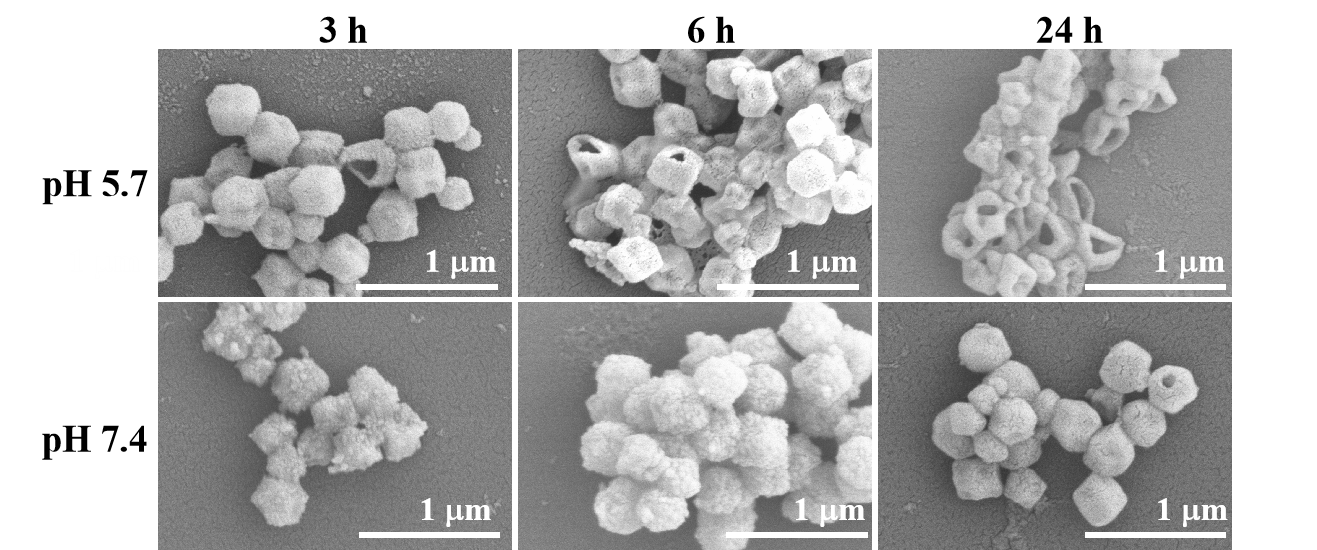
*

***Fig. S3.*** *SEM image of QSZP NPs degraded in PBS (pH=5.7 and pH=7.4) for 3, 6 and 24 h, respectively.*


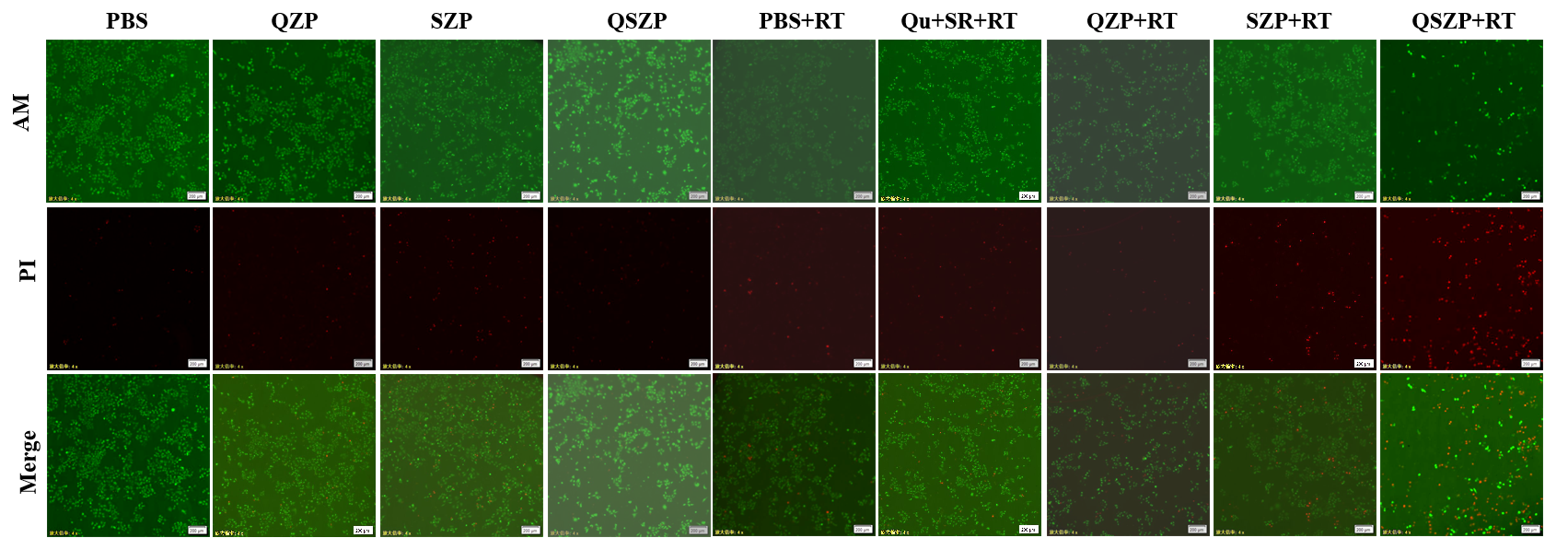


***Fig. S4.*** *Fluorescence images of Calcein-AM and propidium iodide (PI) co-stained in HepG2 cells under different treatments. The scale bar is 200 μm.*


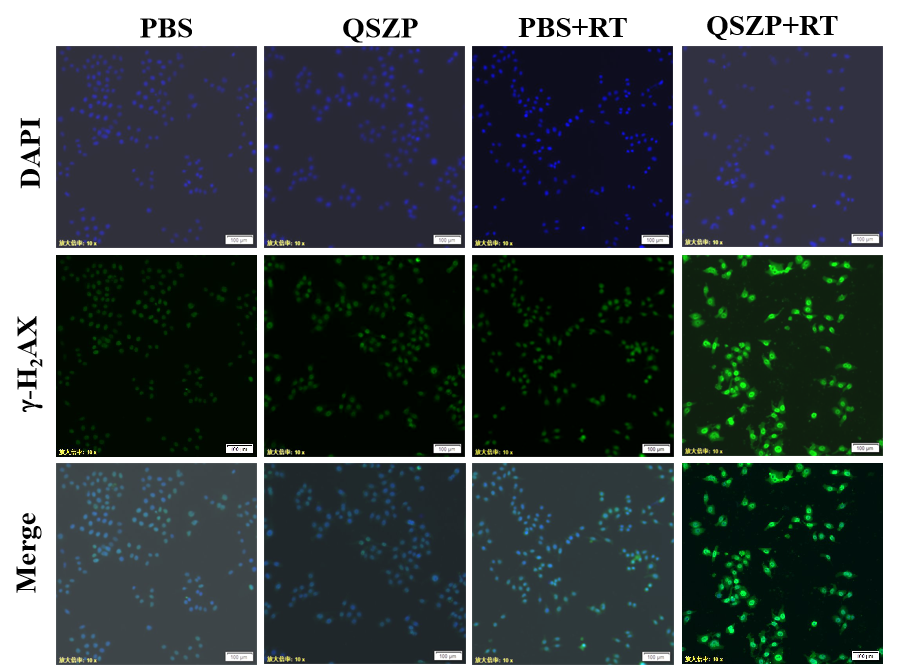


***Fig. S5.*** *Fluorescence images of γ-H_2_AX immunofluorescence in HepG2 cells under different treatments. The scale bar is 100 μm.*

***Fig. S6.*** *The quantitative analysis of γ-H_2_AX fluorescence intensities after various treatments.*

***Table S1*** *Table of tumor volume changes across different treatment groups(mm^3^, n=3, mean ± standard deviation)*

| **Time (day)** | **Control** | **QU+SR+RT** | **QSZP** | **QZP+RT** | **SZP+RT** | **QSZP+RT** |
| --- | --- | --- | --- | --- | --- | --- |
| 1 | 159.30±31.79 | 159.16±12.44 | 159.71±17.84 | 160.38±21.61 | 158.66±20.26 | 159.74±19.33 |
| 4 | 234.53±31.66 | 202.82±19.88 | 246.42±30.76 | 198.41±12.62 | 196.34±40.64 | 183.23±59.09 |
| 8 | 361.96±17.69 | 309.23±6.85 | 321.51±21.58 | 248.42±26.86 | 200.31±50.80 | 140.71±23.22 |
| 11 | 644.04±52.45 | 449.19±33.87 | 464.01±89.66 | 236.52±3.47 | 190.18±18.45 | 96.22±15.71 |
| 15 | 888.72±85.33 | 570.61±29.45 | 580.89±88.32 | 265.88±23.70 | 189.40±4.05 | 65.22±5.49 |
| 18 | 1133.80±136.72 | 698.87±37.62 | 691.51±38.21 | 278.21±29.09 | 219.53±5.26 | 45.62±32.42 |
| 22 | 1309.29±146.45 | 789.90±81.69 | 816.84±36.91 | 321.70±35.02 | 259.92±38.62 | 34.49±59.73 |
| 25 | 1569.45±321.27 | 870.05±35.83 | 975.64±14.18 | 347.08±21.19 | 257.55±16.46 | 37.24±64.51 |


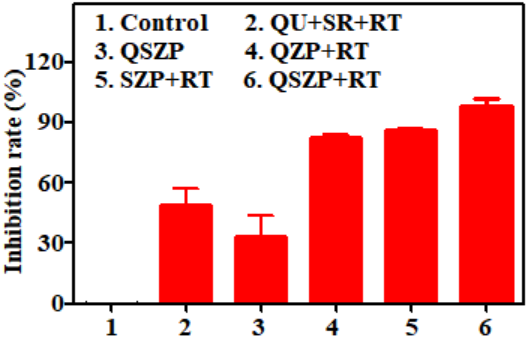


***Fig. S7.*** *Tumor inhibition rates in different treatment groups.*

(Inhibition rate calculation formula: Inhibition rate (%)=(Tumor weight of the control group-Tumor weight of the treatment group)/Tumor weight of the treatment group×100)


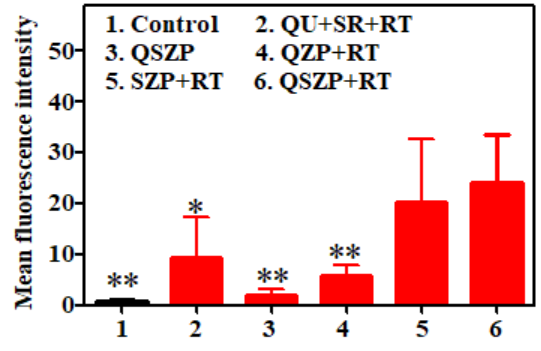


***Fig. S8.*** *The quantitative analysis of FITC fluorescence intensities after various treatments from Fig.7. *P < 0.05 and **P < 0.01, versus the QSZP+RT group.*


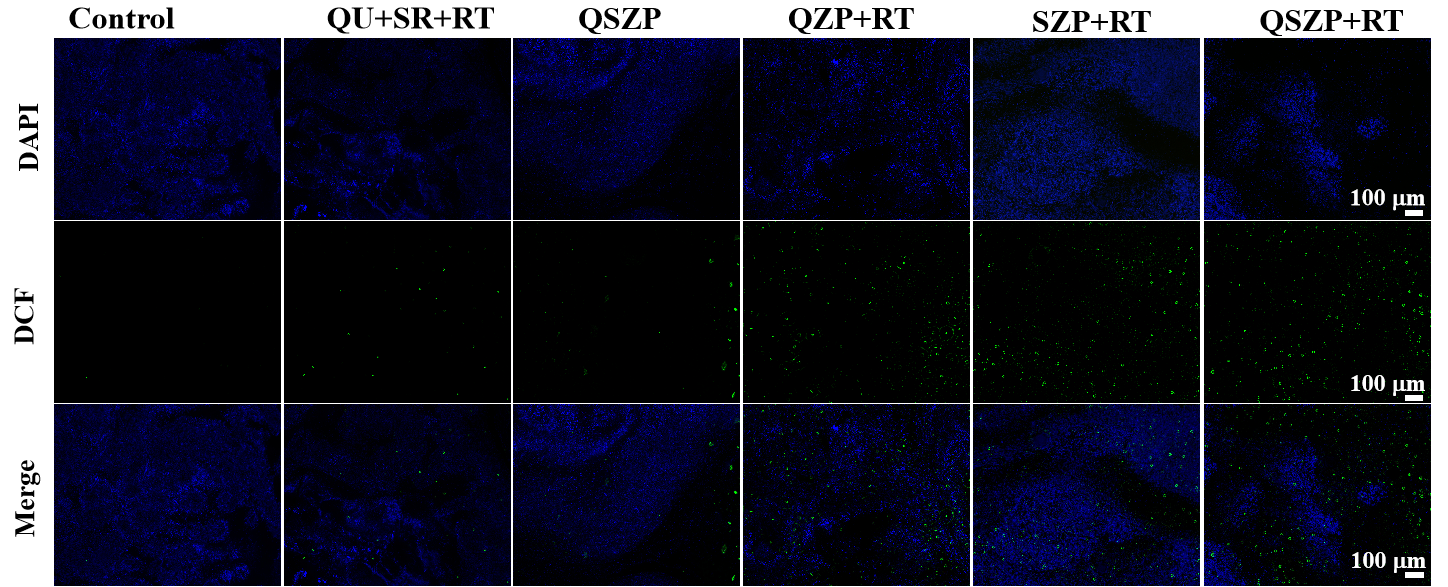


***Fig. S9.*** Inverted fluorescence microscope images of tumor slices after staining with the reactive oxygen species indicator DCFH-DA.


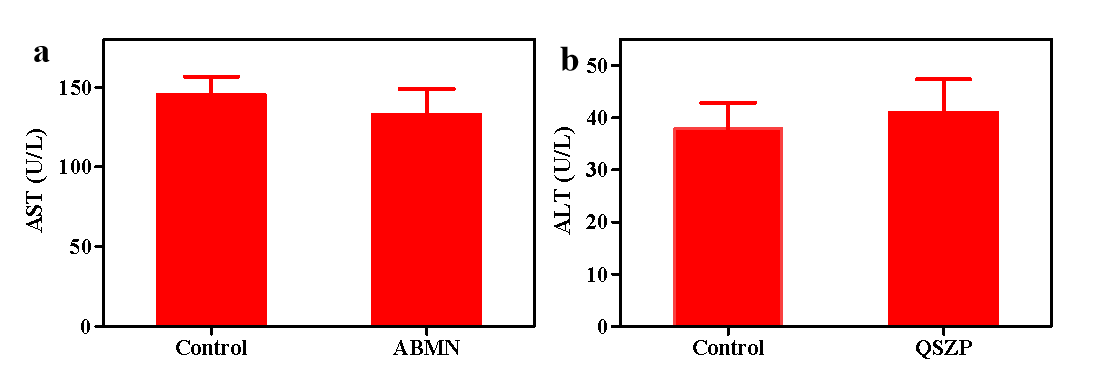


***Fig. S10.*** (a) Aspartate transaminase (AST) and (b) alanine transaminase (ALT) levels recorded for healthy tumor-free Balb/c mice 45 days after iv administration of PBS and QSZP, respectively. Data are presented as mean ± standard deviation (n=3).


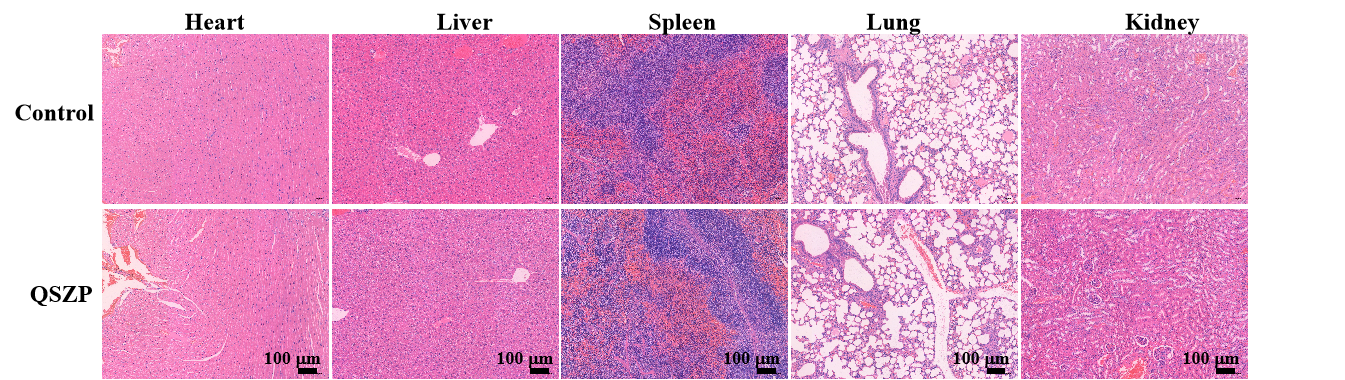


***Fig. S11*** H&E stained tissue sections from the mice to monitor the histological changes in heart, liver, spleen, lung and kidney of the mice 45 days in two groups (n=3).
